# Lower Urinary Tract Symptoms and Overactive Bladder in a Large Cohort of Older Poles—A Representative Tele-Survey

**DOI:** 10.3390/jcm12082859

**Published:** 2023-04-13

**Authors:** Mikolaj Przydacz, Jerzy Gasowski, Tomasz Grodzicki, Piotr Chlosta

**Affiliations:** 1 Department of Urology, Jagiellonian University Medical College, Macieja Jakubowskiego 2, 30-688 Krakow, Poland; 2 Department of Internal Medicine and Gerontology, Jagiellonian University Medical College, Macieja Jakubowskiego 2, 30-688 Krakow, Poland

**Keywords:** lower urinary tract symptoms, overactive bladder, older adults, epidemiology

## Abstract

Background: A reliable reference of population-based parameters for lower urinary tract symptoms (LUTS) and overactive bladder (OAB) is lacking for the elderly. Thus, the objective of this study was to estimate the prevalence, bother, effect on quality of life, and treatment-related behavior for LUTS and OAB in a large population-level cohort of Polish adults aged ≥ 65 years. Methods: We used data from the telephone LUTS POLAND survey. Respondents were categorized by sex, age, and residence. All LUTS and OAB were assessed with validated questionnaires and a standard protocol based on the International Continence Society definitions. Results: The mean (standard deviation) age of 2402 participants (60.4% women) was 72.5 (6.7) years. The prevalence of LUTS was 79.5% (men: 76.6%; women: 81.4%), and the prevalence of OAB was 51.4% (men: 49.4%; women: 52.8%). The prevalence of both conditions increased with age. The most prevalent symptom was nocturia. LUTS and OAB were often bothersome, and almost half of participants who reported LUTS or OAB had decreased quality of life related to their urinary functioning. Nevertheless, only one third of participants sought treatment for their bladder problems, and most of these participants received treatment. We did not observe differences between urban and rural areas in all analyzed population-level parameters. Conclusions: LUTS and OAB were prevalent conditions with significant bother and negative effects on quality of life among Polish adults aged ≥ 65 years. Nevertheless, most affected respondents had not sought treatment. Thus, for older persons, there is a need to increase public awareness about LUTS and OAB, and the negative effects of LUTS and OAB on healthy aging. In addition, greater government and healthcare system resources are needed to better manage LUTS and OAB in older patients.

## 1. Introduction

Lower urinary tract symptoms (LUTS), including overactive bladder (OAB), have negative effects on quality of life and overall wellbeing; thus, LUTS and OAB may significantly limit healthy aging [1]. Although LUTS include diverse groups of symptoms (i.e., storage, voiding, and post-micturition), LUTS are not sex or disease-specific. These symptoms may indicate bladder dysfunction and other structural and/or functional abnormalities of the lower urinary tract, including bladder outlet obstruction. In addition, LUTS may be provoked by a variety of nonurological conditions.

The prevalence of LUTS and OAB increases with age [2]. With aging populations, the number of persons affected by LUTS or OAB and, thus, the effect of these symptoms on functionality and quality of life may increase. For instance, nocturia may lead to interrupted sleep, and drowsiness increasing the risk of falls and fall-related adverse outcome [3,4]. Urinary incontinence, often considered the most bothersome LUTS, significantly affects patient’s self-esteem, mobility, and relationships, and may lead to social isolation [5]. Finally, anticholinergic medications, commonly used for storage LUTS and OAB, may substantially impair the patient’s cognitive status and increase the anticholinergic burden and the long-term risk of the development of dementia [6].

A group of large population-based studies have evaluated the prevalence, bother, and effect on quality of life of LUTS, including OAB [2,7,8]. These epidemiological analyses showed that LUTS were prevalent, often bothersome, and had negative effects on quality of life and overall wellbeing. However, in most of these large population-based studies, investigators did not evaluate exclusively older people in their cohorts; specifically, none of the analyses presented population-level parameters on LUTS/OAB prevalence, bother, effect on quality of life, and treatment behavior dedicated specifically to the elderly. Because the trend of aging-dependent increases in LUTS and OAB prevalence exists, age-specific population-level estimates for LUTS and OAB are needed, and differ from those calculated for the general adult population [9]. The current literature lacks a single reference document of population-level estimates for LUTS and OAB among older people that were reliably obtained with the definitions endorsed by the International Continence Society (ICS) and with validated questionnaires [10]. For high-quality research, it is highly recommended to use generally accepted definitions [11], ideally in representative pools [12]. To promote healthy aging and increase public awareness, it is important to present population-level data for LUTS and OAB dedicated exclusively to older people. Consequently, these data may expedite the development of interdisciplinary and national health programs, with appropriate assignation of healthcare system and government resources. Although there are several community-dwelling and nursing-home studies on LUTS and OAB in the elderly, these data do not reflect population-level parameters. Further, treatment-related behaviors such as treatment seeking, receiving, satisfaction, and continuation among older people may differ from those analysed in the general adult population. As many factors may have an effect on behavior related to health (e.g., culture, ethnicity), treatment preferences associated with LUTS and OAB among old people may also vary between countries and regions. Therefore, in this study, we aimed to assess the prevalence, bother, effect on quality of life, and treatment behavior for LUTS and OAB in a large population-level group of Polish people aged 65 years and older.

## 2. Materials and Methods

This study is a further analysis of data from LUTS POLAND, the first large population-representative, geographically comprehensive (urban and rural), and prospective investigation of LUTS and OAB in a Central-Eastern European country. The concept and detailed methodology for LUTS POLAND have been described in detail elsewhere [13,14].

Briefly, we used data from the latest census (provided by the Central Statistical Office of Poland, Statistics Poland, Polish: Glowny Urzad Statystyczny) and a sample matching technique to produce a representative adult pool of men and women [15]. The survey preserved adequate representation of all 16 provinces (voivodships) in Poland with appropriate ratios of respondents from urban and rural areas. Data collection was conducted by Ipsos Poland (the research agency with proper quality certificates) between 1 September and 30 December 2019. We used computer-assisted telephone interviews; a computerized questionnaire was administered by interviewers to respondents over the telephone. A modified random-digit dialing was employed to randomly select the study participants. Before data collection, we performed a pilot test survey to evaluate linguistic and cultural integrity, and to ensure that lay persons would correctly understand the entire questionnaire. All interviewers were trained and there were regular quality control checks. After data collection, we calculated post-stratification weights to correct any imbalances. The Jagiellonian University Medical College Ethics Committee (1072.6120.160.2019) approved our study, which was also registered with ClinicalTrials.gov (NCT04121936).

### 2.1. Measures

We collected all generic demographic data, including age, sex, marital status, employment status, and level of education. LUTS were evaluated with a standardized protocol based on ICS definitions with storage symptoms (nocturia, frequency, urgency, urinary incontinence), voiding symptoms (intermittency, straining, slow stream, splitting/spraying, hesitancy, terminal dribble), and post-micturition symptoms (incomplete emptying, post-micturition dribble) [10]. The frequency of each specific symptom and the bother of each symptom were evaluated with Likert-like scales (i.e., each frequency question was followed by each bother question; for frequency: none, less than 1 in 5 times, less than half the time, about half the time, more than half the time, almost always; for bother: not at all, a little bit, somewhat, quite a bit, a great deal, a very great deal). We also included questions from the Overactive Bladder-Validated 8-question Screener (OAB-V8), a screening instrument that selects persons with a possible diagnosis of OAB [16], and from the International Prostate Symptom Score (IPSS), the most well-known tool that assesses LUTS severity [17]. With our survey, respondents also provided answers on how their urinary symptoms affected their seeking and receiving treatment, treatment satisfaction, continuation of treatment, and quality of life. All terms and questions were validated and presented in Polish.

### 2.2. Objectives

The prevalence of LUTS in Polish men and women aged ≥ 65 years was the primary objective of this sub-analysis. Investigators who previously analysed LUTS prevalence with population-level surveys used either of two definitions that varied substantially in range of intensity [7,18]. Definition I: at least one storage, voiding, or post-micturition symptom occurring less than half the time or more. Definition II: at least one storage, voiding, or post-micturition symptom occurring half the time or more. The presented approach enabled us to make comparisons with other studies in which LUTS were analysed at the population level. In addition, before initiating the survey, we pre-specified the objective of this sub-analysis in the study design of LUTS POLAND.

Secondary study objectives were to document the prevalence of LUTS in different age groups (5-year intervals), the prevalence of specific LUTS, the sex differences in specific LUTS, the bother of specific LUTS (an answer “quite a bit” was a threshold for bother), the prevalence of LUTS subgroups, the prevalence of OAB (score  ≥ 8 points from the OAB-V8), the overall severity of LUTS (based on the IPSS), the effect on quality of life, and the behavior related to LUTS and OAB treatment (treatment seeking, receiving, satisfaction, continuation).

### 2.3. Statistics

Different methods of descriptive statistics were used to present demographic variables and assess the initial data. The Kruskal–Wallis test was used for continuous variables, and the Chi-squared test for categorical variables. A *p* value < 0.05 was considered statistically significant. For all data analyses, we employed SPSS Statistics software (IBM, version 24.0, IBM Corp., Armonk, NY, USA).

## 3. Results

Of 6005 respondents from throughout Poland who completed the LUTS POLAND survey, 2402 participants were 65 years of age or older. There were more women than men (60.4% vs. 39.6%, *p* < 0.01). The mean age for the entire cohort was 72.5 ± 6.7 years. Because our sample size was large, the respondents were categorized into six age groups, 65–69, 70–74, 75–79, 80–84, 85–89, and ≥90 (39%, 31%, 15%, 10%, 3%, 2%, respectively; percent of all participants in this sub-analysis). More participants were residing in urban than in rural regions (67% vs. 33%, *p* < 0.01).

### 3.1. The Prevalence of LUTS

**Figure 1:**
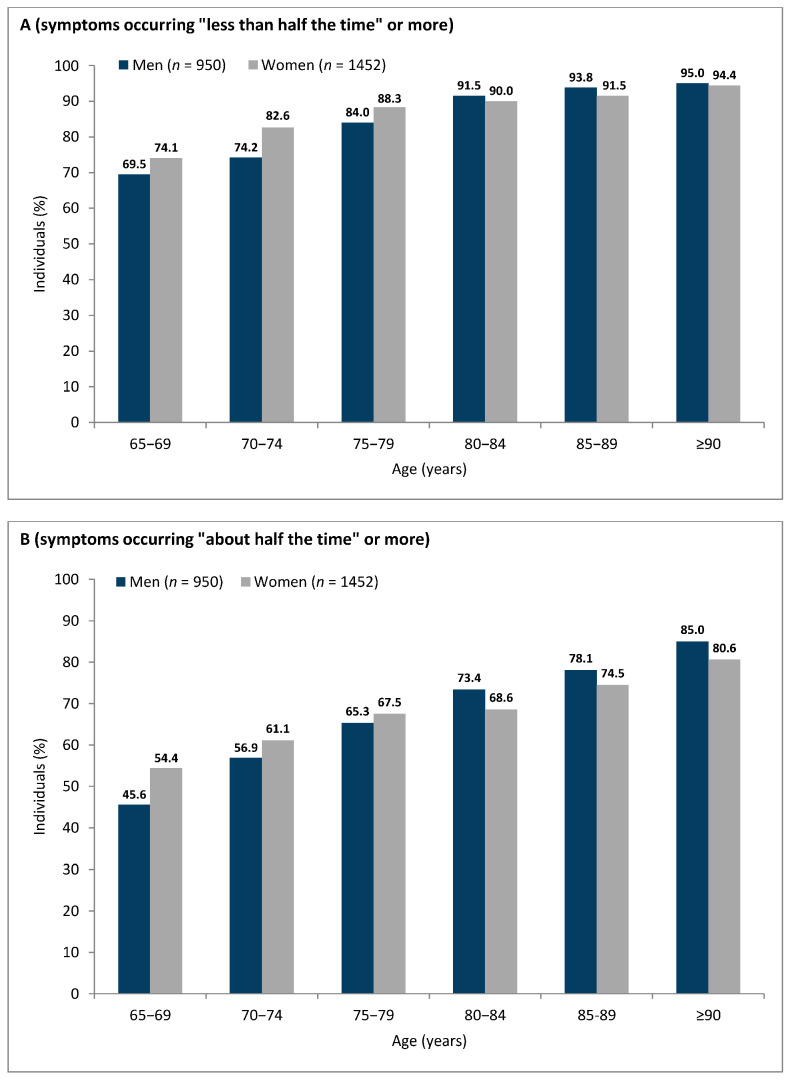
Prevalence of lower urinary tract symptoms in persons aged ≥ 65 with the two study definitions: (**A**) definition I—symptoms occurring “less than half the time” or more; (**B**) definition II—symptoms occurring “about half the time” or more.

Overall, the prevalence of at least one LUTS at least ‘less than half the time’ (definition I) in persons aged ≥65 years was 79.5% (men: 76.6%; women: 81.4%; *p* < 0.01). The prevalence of at least one LUTS at least ‘half the time’ (definition II) in our cohort was 59.3% (men: 56.6%; women: 61.0%; *p* < 0.05). Regardless of the definition, the prevalence of LUTS showed an increasing trend with age for both men and women (*p* < 0.05; Figure 1). In younger respondents (i.e., age groups 65–69, 70–74, 75–79), LUTS were more prevalent in women than in men. In older respondents (i.e., age groups 80–84, 85–89, ≥90), LUTS were more prevalent in men than in women (this trend was observed with both definitions of LUTS prevalence, Figure 1). We did not find any correlations between LUTS prevalence with urban/rural status or across geographical regions (voivodships) of Poland.

### 3.2. The Prevalence and Bother of Specific LUTS

Nocturia was the most prevalent symptom of both men (definition I: 50.8%, definition II: 26.2%) and women (definition I: 52.8%, definition II: 28.0%; Table 1), followed by frequency (for men, definition I: 33.2%, definition II: 24.8%; for women, definition I: 35.5%, definition II: 26.7%).

Considering sex differences, women reported significantly more often urgency with fear of leaking, urge urinary incontinence, stress urinary incontinence, mixed urinary incontinence, and leak for no reason (Table 1). Importantly, all these symptoms are categorized as storage symptoms. On the other hand, men reported significantly more often intermittency, hesitancy, straining, slow stream, splitting/spraying, and terminal dribble. Particularly, all these symptoms are voiding. There were no geographical and urban/rural differences in prevalence of specific symptoms.

The most bothersome symptom for men was urgency with fear of leaking, followed by mixed urinary incontinence (Table 1). For women, stress urinary incontinence was the most bothersome symptom, followed by leak for no reason. Overall, storage symptoms tended to be more bothersome than voiding or postmicturition symptoms for both men and women.

### 3.3. The Prevalence of LUTS Subgroups

In our large cohort of respondents aged 65 years and older, storage symptoms encompassed the predominant ICS symptom group (definition I: 65.8% overall, 63.9% of men, 67.0% of women; definition II: 60.6% overall, 59.5% of men, 61.3% of women). Voiding symptoms were reported by 31.2% of respondents according to definition I (43.2% of men; 23.4% of women) and 20.8% according to definition II (30.1% of men; 14.7% of women). A group of postmicturition symptoms had the lowest prevalence (definition I: 14.4% overall, 20% of men, 10.7% of women; definition II: 8.1% overall, 11.4% of men, 5.9% of women).

### 3.4. The Prevalence of Overactive Bladder

With the OAB-V8 questionnaire and a threshold of at least 8 points in this instrument, we estimated that 51.4% of the respondents aged 65 years and older might have OAB. Considering sex differences, OAB was slightly more prevalent in women than in men (52.8% vs. 49.4%).

### 3.5. The Overall Assessment of Severity of LUTS

According to the IPSS categories (none, mild, moderate, severe), we observed that most of the study participants reported mild symptoms (score 1–7; 57.2% of men, and 68.7% of women). The remaining respondents presented moderate symptoms (score 8–19; 31.1% of men, and 21.7% of women), no symptoms (score 0; 6.2% of men, and 7.4% of women), and severe symptoms (score 20–35; 5.5% of men, and 2.2% of women).

### 3.6. Quality of Life

On the basis of the question: “If you were to spend the rest of your life with your urinary condition just the way it is now, how would you feel about that?”, we denoted that LUTS in the older population had a significant, negative impact on quality of life. With definition I, we observed that 38.9% of the persons with LUTS responded ‘mixed’, ‘mostly dissatisfied’, ‘unhappy’, or ‘terrible’. By definition II, 49.8% of the persons responded similarly. The negative effect of LUTS on quality of life was comparable between men and women, regardless of the LUTS prevalence definition.

### 3.7. Behavior Related to Treatment

Only one-third (31.4%, *n* = 533) of respondents with at least one LUTS at least ‘less than half the time’ (definition I) sought treatment, and most of these individuals obtained treatment (26.2%, *n* = 444). Among participants who reported at least one LUTS at least ‘half the time’ (definition II), one-third (33.1%, *n* = 510) sought treatment, and most received treatment (29.2%, *n* = 450). Men more often sought treatment for LUTS than women (definition I: *n* = 271, 38.4% of men, *n* = 262, 26.4% of women; definition II: *n* = 261; 39.9% of men, *n* = 249, 28% of women; *p* < 0.01). The majority of participants who received treatment were satisfied with their therapy (definition I: 59.4%; definition II: 64.9%). We did not find any disparities between treatment seeking/receiving/satisfaction, urban/rural areas, and geographical regions (voivodships) of Poland.

## 4. Discussion

The concept of healthy aging was proposed by the World Health Organization, and is defined as “the process of developing and maintaining the functional ability that enables wellbeing in older age” [19]. The functional ability was further discussed as an individual’s ability to meet basic needs, learn, grow, make decisions, be mobile, build and maintain relationships, and contribute to society. Therefore, with this concept, the elderly can expect support in creating the environments and opportunities that empower their optimal wellbeing, particularly in countries with relatively long life expectancy. Our study is the first epidemiologic assessment of LUTS and OAB in older Poles, conditions that can significantly limit healthy aging.

In our large cohort of older men and women, LUTS and OAB were highly prevalent and bothersome. Despite the fact that these conditions often had a negative effect on quality of life, treatment seeking for LUTS and OAB was low. Because LUTS and OAB in older persons are associated with poorer health status and physical impairment that limit healthy aging, these symptoms are a significant public health issue that requires special attention at the central level of public healthcare systems. Moreover, because of population aging around the world, the burden of LUTS and OAB will continue to increase.

The continuation of the LUTS POLAND study that we show in this paper is the population-level analysis of LUTS and OAB that specifically addressed these symptoms in an exclusive cohort of older persons. The analysis expands the knowledge of overall and sex-specific LUTS and OAB population rates in individuals aged 65 years and older. In addition, the data that were used for this study came from a one-country, nationally representative group of adults, and were obtained with validated instruments and generally accepted definitions. Therefore, this report with reliable population-level parameters for LUTS and OAB may serve as a reference document that will direct future, particularly prospective, studies on LUTS and OAB and their management for older patients.

The prevalence of LUTS and OAB have been studied in a number of surveys, which yielded disparate results, with the overall LUTS prevalence varying from 10% to 90% [9,20], and the overall OAB prevalence from 2% to 53% [21]. Most of these studies were lacking in the analyses pertaining to older adults, and often simply extrapolated the prevalence in younger cohorts to persons aged ≥80 years [21]. Studies of older, community-dwelling persons were not typically based on generally approved ICS definitions, but instead, were often based on simple, unvalidated questionnaires [22,23,24]. Importantly, the variability between questionnaire-defined and ICS-defined LUTS prevalence still exists. Various instruments (e.g., IPSS) enquire differently about LUTS. The questions and response options might be differently interpreted and, consequently, result in differences between studies. Further, translation into multiple languages may also extend variability when not supported by proper cultural and clinical validation. In our study which was conducted with ICS definitions and validated questionnaires, we showed that LUTS occurring ‘less than half the time or more’ affected 79.5% of persons aged 65 years or older, and LUTS occurring ‘half the time or more’ affected 59.3% of that group. In addition, we estimated that 51.4% of the respondents aged 65 years or older have OAB. Because our data originated from a representative group of adults from a highly homogeneous population, our findings may be considered as an estimate of the global scope of LUTS and OAB in older persons.

The study participants often reported LUTS and OAB as bothersome with negative effects on quality of life. Nonetheless, only one third of respondents had sought treatment. Because LUTS and OAB may significantly limit healthy aging, our observation is especially disquieting. The low frequency of seeking treatment for LUTS and OAB has been documented in some earlier analyses. Our data confirm and expand this finding. [25]. LUTS and OAB are significantly associated with lower functional ability, fatigue, and symptoms of depression [1]. LUTS and OAB may be ignored and left without proper management because of assumptions that the conditions are the natural consequences of aging, regardless of social environment. All these observations imply that there is a strong need to raise public awareness of LUTS and OAB in older patients. The lack of knowledge of treatment options may cause a lack of searching for medical advice. Importantly, the role of primary care physicians should not be dismissed, as they can screen for LUTS and OAB, and start simple diagnostics together with the initiation of noninvasive treatments (e.g., lifestyle changes and behavioral interventions). Further, greater healthcare resources for LUTS and OAB in older patients are also needed because these individuals should obtain a holistic multidisciplinary approach for their urinary functioning. As the etiology of LUTS and OAB in older people is multifactorial and includes physical and cognitive disabilities, comorbidities, surrounding environment, impaired conscious willingness, and often underlying neurological disorders, the identification of LUTS in this specific cohort of patients may be more complex than one would expect. It is clear that close cooperation is required between different medical specialties, including urologists, gynecologists, and geriatricians, in particular. In addition, as the management for LUTS and OAB may be conservative, pharmacological, and surgical, optimal financial resources are needed to overcome barriers related to the availability of treatment methods and new treatment technologies.

In our analysis, urban vs. rural residence did not impact the search for, and the supply of, medical help for LUTS and OAB. When we were planning our study, we hypothesized that people who lived in rural regions would be less active in seeking treatment for their urinary dysfunctions compared to people in urban regions. The hypothesis originated from a study by Branowitzer, who showed that persons from Polish urban regions were less hesitant to report their health problems [26]. However, these data are now decades old. Our new data indicate that healthcare delivery differences between Polish rural and urban areas are no longer extant. In addition, we should consider that Polish urban and rural regions have begun to merge as the population density in Poland has been increasing [27].

The study has several limitations. The participants self-reported their symptoms without medical evaluation to measure LUTS or, importantly, OAB. In daily practice, OAB can be diagnosed in the absence of urinary tract infection or other obvious pathology that can lead to storage symptoms. Even though we used a validated OAB screening tool (OAB-V8), we could not exclude storage symptoms secondary to other conditions unrelated to OAB. Cold-calling may lead to the failure to report sensitive issues like those related to bladder problems. In addition, we did not ask about comorbidities and did not consider the effects of polypharmacy. However, it would be considerably difficult to reliably confirm all relevant comorbidities and drugs taken from a self-reporting respondent interviewed by telephone. This limitation of population-based self-reported data has been extensively described by Coyne et al. [28]. Because this was a telephone survey, we also did not reliably assess the cognitive function of the respondents. However, we conducted pilot telephone-surveys and cognitive debriefing interviews before we started final data collection. This pilot phase confirmed that lay persons would correctly understand all questions. Finally, as our analysis was performed only in Poland, some of the presented findings may not be generally applicable to other populations of older adults, e.g., for treatment-related behavior. However, our results are comparable with those presented from other countries that also showed low rates of treatment seeking for LUTS or OAB [29].

## 5. Conclusions

LUTS and OAB are prevalent conditions among older Poles. LUTS and OAB are often troublesome, and they impair quality of life. Nevertheless, most affected individuals seldom seek treatment. Thus, it is essential to execute and promote strategies to raise public awareness about LUTS and OAB, and their negative effects on healthy aging.

## Figures and Tables

**Table 1:** Prevalence of specific symptoms according to definition I (symptoms occurring less than half the time or more) and definition II (symptoms occurring about half the time or more) and associated bother in men and women aged 65 years or older.

	Men (*n* = 950)						Women (*n* = 1452)					
	Symptom Prevalence (Definition I)		Symptom Prevalence (Definition II)		Prevalence of Bother (At Least Quite a Bit) ^a^		Symptom Prevalence (Definition I)		Symptom Prevalence (Definition II)		Prevalence of Bother (At Least Quite a Bit) ^a^	
	*n*	%	*n*	%	*n*	%	*n*	%	*n*	%	*n*	%
Storage symptoms												
Nocturia ^b^	483	50.8	249	26.2	142	57.0	767	52.8	407	28.0	231	56.8
Frequency	315	33.2	236	24.8	123	52.1	516	35.5	388	26.7	211	54.4
Urgency	225	23.7	129	13.6	104	80.6	359	24.8	219	15.1	181	82.6
Urgency with fear of leaking	141	14.8 *	98	10.3 **	93	94.9	308	21.2	270	18.6	231	85.6
Urge urinary incontinence	61	6.4 **	31	3.3 ***	25	80.6	201	13.8	132	9.1	117	86.6
Stress urinary incontinence	33	3.5 ***	20	2.1 ***	17	85.0	210	14.5	125	8.6	117	93.6
Mixed urinary incontinence ^c^	25	2.6 ***	16	1.7 ***	15	93.8	205	14.1	112	7.7	96	85.7
Leak for no reason	38	4.0 *	19	2.0	17	89.5	96	6.6	52	3.6	46	88.5
Voiding symptoms												
Intermittency	164	17.3 **	110	11.6 **	73	66.4	139	9.6	82	5.6	53	64.6
Slow stream	265	27.9 **	162	17.1 **	99	61.1	187	12.9	94	6.5	50	53.2
Hesitancy	138	14.5 **	71	7.5 **	55	77.5	95	6.5	42	2.9	32	76.2
Straining	97	10.2 ***	59	6.2 ***	50	84.7	39	2.7	16	1.1	10	62.5
Splitting/spraying	136	14.3 **	71	7.5 **	41	57.7	81	5.6	42	2.9	27	64.3
Terminal dribble	237	24.9 **	158	16.6 **	98	62.0	172	11.8	104	7.2	63	60.6
Post-micturition symptoms												
Incomplete emptying	162	17.1 **	94	9.9 **	77	81.9	116	8.0	62	4.3	45	72.6
Post-micturition dribble	80	8.4	42	4.4	35	83.3	65	4.5	36	2.5	30	83.3

^a^ Prevalence of bother was based on definition II. ^b^ Nocturia was defined as two or more voids per night. ^c^ Participants who reported both urge and stress urinary incontinence symptoms were classified as having mixed urinary incontinence. * *p* ≤ 0.05, men vs. women. ** *p* ≤ 0.01, men vs. women. *** *p* ≤ 0.001, men vs. women.

## Data Availability

All data generated or analysed during this study are included in this published article.
